# Annotations

**Published:** 1896-10-31

**Authors:** 


### Annotations.

### "Levelling Up the Villages."

When a body like the Incorporated Law Society takes upon itself to discuss the condition of our English villages, not entirely from a legal, but almost more from a sanitary and humanitarian point of view, those physiologists who make a study of "race development" and "race progress" may well begin to hold up their heads a little. The medical and sanitary professions have had an uphill fight for at least half a century to persuade the other classes of the community that farm labourers, alike from the economic and the sanitary standpoints, ought to be scientifically considered by the statesman who desires to keep himself abreast of the times. A feeble and dissatisfied peasantry is a serious encumbrance, and may become an equally serious danger. It is not difficult to make farm labourers "healthy and wealthy and wise." They are probably more responsive to rational treatment than any other members of the industrial class. Three conditions need to be fulfilled in order to make them vigorous in body and contented in mind. They must be provided with good house accommodation, on healthy sites, and surrounded by abundance of air and light; they must be further provided with a little land in which they may employ their spare hours, and, above all, eke out their somewhat scanty resources; and they must be furnished with an elementary education. If all these things be gradually done for them all, as they are being done for some, the charms of the country and of an open-air life may be depended upon to do the rest, and to keep the nation supplied with a healthy peasantry, and a race of men fit for soldiering and national defence. Medicine has taught the nation these elementary truths for many years. She continues to insist that nothing will pay the nation better than health among its poor. She urges that all villages and hamlets should, with all speed, be levelled up to the condition in things sanitary and economic which many villages have reached; and she looks forward to a time when England, as to her rural population, will be "merrier" than ever she was in the long past, because she will be healthier and more prosperous in every detail of life.

### The Hospital Reform Association.

A circular issued by Dr. Ward Cousins and Mr. Garrett Horder, and purporting to be from the "Hospital Reform Association," lies before us. It may be presumed that the circular invites criticism; and the most obvious of all criticisms is this—"First catch your association." For we can hardly believe that the association is formed and developed, otherwise the circular would at least have given us the names of the members of its governing body. In these days, and especially in the medical profession, we like to know who it is that claims our suffrages; who it is that proposes to reform our institutions; and, above all, who it is that asks us for annual subscriptions and personal membership, even if it be only to the extent of giving five shillings and one's name. Let us, then, in the first place, see the association in its personnel; that is, as far as possible, let us look it squarely in the face in the flesh. Now, in the second place, as to "proposals" for reform. What are they? They seem to consist almost, if not entirely, of the old methods; and they are not even trotted out under new names. The subject of hospital reform is to be "ventilated." By all means; only we thought it had already had every variety of "ventilation" tried upon it. The "ventilation" is to be carried out by means of "papers and pamphlets." The faith of inexperienced mortals in "printed matter" is touching, almost to sublimity. Papers and pamphlets! Who is to write them? Above all, who is to read and act upon them? The next step is to "make representations" to "hospital managers and members of the hospital staff." Alas, alas! What a task is here for somebody! Are the "representations" to be printed or personal? If printed, we see in a vision unnumbered waste-paper baskets visibly expanding and swelling to the bursting point. If personal, we hear by anticipation the polite "Good morning" with which the "representers" will be bowed out by the "hospital managers and members of the hospital staff." In truth, we are sincerely sorry for the new association. A fellow-feeling makes us wondrous kind. The labours of Sisyphus are nothing to the task it has undertaken! Do we, then, despair of hospital reform? Have we arrived at no decision as to its necessity? On the contrary, we are convinced of its necessity, and we hope for its achievement. But when we see an association started for this object, consisting, as it appears, almost entirely of medical men, and those, presumably, for the most part busy general practitioners, then we know at the outset that such an association is foredoomed to complete and utter failure. We sympathise most deeply with the objects of the new association; but if ever those objects are to get beyond the "paper and pamphlet" stage, there must be a new beginning and a differently constituted association.

### The Serum Treatment of Diphtheria.

It is very satisfactory to hear of the advances which have recently been made in the treatment of diphtheria by anti-toxic serum. Dr. Sidney Martin, speaking of the results obtained in University College Hospital, says that there has undoubtedly been a saving of life in children under five years of age, and that the better results in 1896 as compared with 1895 are to be ascribed to the better serum used. This is especially gratifying, because what is here spoken of as "better" serum, really is a stronger serum, and with every increase of strength it is possible to administer the necessary dose with a smaller admixture of other animal substances. It appears that the serum recently supplied from the laboratories of the Royal Colleges of Physicians and Surgeons by Dr. Sims Woodhead is eight times as strong as some other samples of serum which are in use. Hitherto the expense of producing this potent serum has been very great, for only a few horses are capable of yielding it, and much labour and months of time have had to be expended in the comparatively preliminary work of testing the serum-producing power of the horses. By the methods now adopted, however, this can be done in a much shorter time, and the horses that are found incapable of yielding a serum of high potency are quickly got rid of and replaced by others. It is hoped that before long these potent serums will be available for general use at a moderate price.

